# Biliary atresia with an unusual abdominal orientation: A case report

**DOI:** 10.1016/j.ijscr.2019.01.028

**Published:** 2019-01-30

**Authors:** Jawad Allarakia, Taher Felemban, Waleed Khayyat, Ahmed Alawi, Abdurrahaman Mirza, Batool Alkhazal, Yasmin Yousef

**Affiliations:** a King Saud Bin Abdulaziz University for Health Sciences, College of Medicine, Jeddah, SAudi Arabia; b Department of Surgery, Pediatric Surgery Section, King Fahad Armed Forces Hospital,Jeddah, Saudi Arabia; c Departmetnt of Surgery, Pediatric Surgery Section, King Abdulaziz Medical City, Ministry of National Guard Health Affairs, Jeddah, Saudi Arabia; d College of Medicine – Jeddah, King Abdulaziz University, Jeddah, Saudi Arabia; e King Abdullah International Medical Research Center, Jeddah, Saudi Arabia

**Keywords:** Biliary atresia, Case report, Congenital abnormalities, Situs anomalies, Heterotaxy

## Abstract

**Introduction:**

Biliary atresia (BA) is a rare condition where the hepatic or common bile ducts get obliterated by fibrous tissue for unknown causes. It is diagnosed in 5–10 per 100,000 live births worldwide in whom liver injury and death are definite if untreated.

**Presentation:**

We report a case of BA with associated malrotation and situs ambiguous discovered incidentally during Kasai procedure. The small intestine was found to be malrotated with the duodenojejunal junction to the right of the vertebral column. The liver, stomach, and spleen were in the center, right side, and left side of the abdomen respectively. The malrotation was corrected by performing a Ladd’s procedure in addition to the Kasai. She had an uneventful postoperative course. At follow-up on the second, sixth and 12th weeks postoperatively, she had normal stool color and liver function.

**Discussion:**

BA patients with associated structural anomalies might have a worse outcome after the Kasai procedure. Several studies demonstrated that the coexistence of malrotation with BA to be in the range of 0.3%–9.4% among BA patients. Also, the presence of situs abnormalities with BA has been reported in some studies to be present in 0.01–0.045% of BA cases. However, the presentation of BA with both situs inversus and intestinal malrotation has seldom been reported.

**Conclusion:**

The diagnosis of BA should prompt further investigation for other anatomical abnormalities as the presence of which might affect the management plan.

## Highlights

• Biliary atresia is a rare condition that causes jaundice in neonates.
• An association between biliary atresia and other developmental abnormalities has been reported.
• A presentation of biliary atresia with intestinal malrotation and situs ambiguous is extremely rare.
• The diagnosis and surgical management of such cases require meticulous evaluation.
• The use of post-operative steroids remains controversial.

## 1 Introduction

Biliary atresia (BA) is a rare condition where the hepatic or common bile ducts get obliterated by fibrous tissue for unknown causes. It is diagnosed in 5–10 per 100,000 live births worldwide in whom liver injury and mortality are definite if left untreated [1,2]. Since 1929, when a report suggested an association between BA and polysplenia, several anatomical anomalies have been reported to coexist with BA [[3], [4], [5], [6]]. The introduction of Kasai procedure in 1959 has offered patients with BA long-term survival. However, it is suggested that patients with associated structural anomalies might have a worse outcome after the procedure [7]. In this report, we describe an unusual presentation of BA with associated malrotation and situs ambiguous, which were discovered incidentally during the Kasai procedure. This work has been reported in line with the SCARE criteria [8].

## 2 Case presentation

This is a full-term female child born by spontaneous vaginal delivery weighing 2.08 kg. The baby is the third child to her nonconsanguineous parents after two sons aging six years and three years, both of which are healthy.

The child developed physiological jaundice right after birth. It resolved with phototherapy. The child remained healthy until the age of 45 days when she presented to the emergency room with a one-day history of fever that improved with paracetamol. Physical examination revealed a sick-looking jaundiced baby with no dysmorphic features. Examination of the abdomen demonstrated a soft nontender abdomen, with a reducible umbilical hernia. No hepatosplenomegaly was noted at that time. The patient was then admitted for further evaluation of her jaundice. On admission, the patient’s stool and urine color were normal, but soon after, her stool started to become paler in color.

Initial laboratory tests included complete blood count, coagulation profile, kidney function tests, all of which were within normal ranges. However, total bilirubin, direct bilirubin, and liver enzymes were elevated [total bilirubin: 296.7 (0.8–6.8) μmol/L, direct bilirubin: 208.50 (0.8–3.4) μmol/L, AST: 24.86 (0.37-0.73) μmol/L, Alk-Ph: 13.47 (2.6–369) μmol/L, ALT: 7.13 (0.15–0.42) μmol/L, and GGT: 1.95 (0.1–0.27) μmol/L].

An abdominal ultrasound showed a small gallbladder measuring 1.3 cm in its longitudinal parameter. However, the common bile duct was not visualized. These findings were highly suspicious for BA. At that time, a liver biopsy revealed fibrosis Stage I, and moderate portal inflammation. These findings were consistent with the diagnosis of extra-hepatic BA, but were also suggestive of neonatal hepatitis as an alternative differential diagnosis. Intraoperative cholangiogram was planned without a HIDA scan because the patient was approaching day 60 of age. Further delay would have risked a suboptimal outcome of a Kasai procedure [9,10].

At the age of 65 days, the patient was taken for intra-operative cholangiogram and to proceed to a Kasai procedure if indicated. At the time of surgery, a small gallbladder and absent biliary tree were revealed. These findings, confirmed the diagnosis of BA. Thus, the abdominal incision was extended to perform the Kasai procedure.

**Fig. 1:**
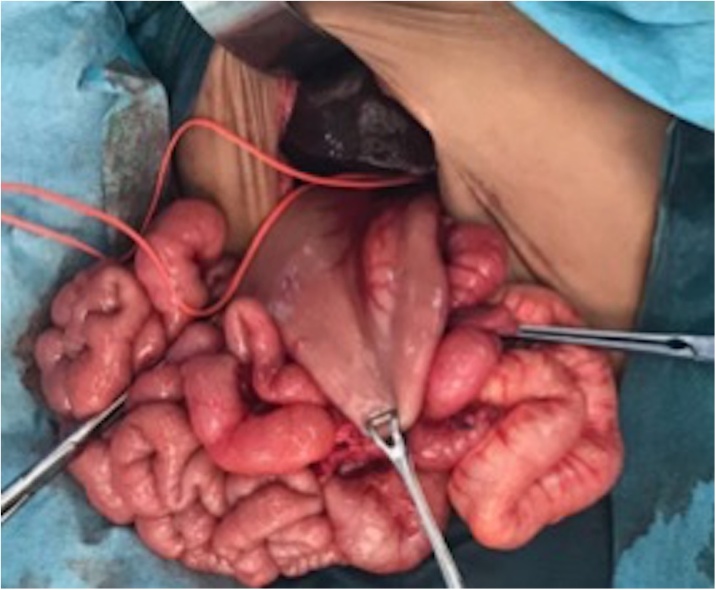
Abdominal Orientation: Intraoperative photo showing the stomach on the right side of the abdominal cavity and the liver is centralized.

During the procedure, the liver was found to be at the center of the abdomen, which required the extension of the surgical wound. The stomach was located in the right side of the abdominal cavity lateral to the liver (Fig. 1). In addition, malrotation was identified with the duodenojejunal junction to the right of the vertebral column. The cecum and appendix were in the left upper quadrant, and a globular spleen was found on the left side. The malrotation was then corrected by performing Ladd’s procedure. Then a Roux loop was taken 40 cm distal to the duodenojejunal junction and an end-to-side portoenterostomy was performed (Kasai).

The patient was kept *Nil Per Os* (NPO) for five days, after which she was started on breast feeding which was well-tolerated. She passed green-colored stool on the fourth post-operative day. She was also started on intravenous Methylprednisolone postoperatively at a dose of 2.6 mg/kg/day twice a day for five days. After which, she was shifted to oral prednisone once daily. The patient was discharged on the seventh post-operative day on prednisolone, ursodeoxycholic acid, multivitamins with vit. A, D, E and K drops, and oral trimethoprim/sulfamethoxazole antibiotics 20 mg twice a day for 15 weeks.

She was seen for follow-up on the second, sixth and 12th week post-operatively in the pediatric surgery and gastroenterology clinics. Upon follow-ups, stool became normal in color and liver function test results continued to normalize throughout that period. By the fourth month postoperatively, the liver function test were as follows total bilirubin 1.9 μmol/L, AST 0.6 μmol/L, Alk-Ph 4.75 μmol/L, and ALT 0.45 μmol/L.

## 3 Discussion

Biliary atresia is a progressive fibrosis and obliteration of the hepatic biliary tree. In spite of the fact that it is one of the most common neonatal cholestatic disorders, its etiology is still not clearly understood. Jaundice usually appears after two weeks of birth, sometimes in continuity after physiologic jaundice adding to the confusion. Although BA is known to coexist with different anatomical variations, studies have varied results on the prognosis of isolated BA when compared with BA that is associated with congenital anomalies [[3], [4], [5], [6]].

One of the known associated anomalies is intestinal malrotation, which is a range of developmental anomalies occurring during fetal development resulting in abnormalities in the position of the small and large intestines and their attachments to the peritoneum [11]. It was demonstrated that up to 12.1% of BA have coexisting malrotation [12].

Another associated structural variation is situs anomaly. It can be further defined as situs inversus or situs ambiguous. Situs inversus is a rare congenital malformation where the internal organs are mirrored, whereas situs ambiguous is not defined with a characterized set of organs location but rather is a spectrum of anatomical abnormalities. Situs anomalies and intestinal malrotation are known to coexist. Also, the presence of situs abnormalities with BA has been reported [13]. However, up to our knowledge, the presentation of BA with both situs inversus and intestinal malrotation has only been reported three times [[14], [15], [16]].

Previous studies suggest that around half of the patients that have had a Kasai procedure will eventually need liver transplantation within 20 years [17,18]. Moreover, the outcome of Kasai procedure has been shown to be affected by different factors. For example, an important prognostic factor is the patient’s age at operation. Studies suggested that performing the procedure before 60 days of age has preferable outcomes, where others suggest an extended period of 100 days [9,10,19].

Another factor that is believed to predict a poorer prognosis is having associated anomalies with BA [7]. However, due to the limited number of cases, the data on prognosis is not adequately established. In addition, some other reports found no statistical difference in the overall survival [13].

Our patient had multiple congenital malformations and had the procedure at 65 days of age. She continued to show very good laboratory and clinical improvement. We are hopeful that she will continue to have a good course.

## 4 Conclusion

As described in the literature and in our case, the diagnosis of BA should indicate further evaluation for other anatomical abnormalities for its potential importance in altering treatment plans and the surgical approach.

## Conflicts of interest

None.

## Sources of funding

This research did not receive any specific grant from funding agencies in the public, commercial, or not-for-profit sectors.

## Ethical approval

Ethical approval was obtained from King Abdullah International Medical Research Center, the institutional review board in King Abdulaziz Medical City in Jeddah.

## Consent

Written informed consent was obtained from the patient’s father for publication of this case report and accompanying images. A copy of the written consent is available for review by the Editor-in-Chief of this journal on request.

## Author’s contribution

- Jawad Allarakia: Literature review, Acquisition of data, Writing the article.
- Taher Felemban: Literature review, Writing the article.
- Waleed Khayyat: Literature review, Writing the article.
- Ahmed Alawi: Writing the article, Patient care and Follow up.
- Abdurrahaman Mirza: Literature review.
- Batool Alkhazal: Literature review.
- Yasmin Yousef: Consultant treating the patient, Study idea, Interpretation of data, Critical revision of the article for intellectual content, Final approval of the submitted version.

## Registration of research studies

This is not a ‘first in humans’ report, so it is not in need of registration.

## Guarantor

Dr. Yasmin Yousef, Departmetnt of Surgery, Pediatric Surgery Section, King Abdulaziz Medical City, National Guard Health Affairs, Mail Code 6636 P.O. Box 9515 Jeddah, 21423, Saudi Arabia.

## Provenance and peer review

Not commissioned, externally peer-reviewed.
